# Surgical treatment of thoracolumbar fracture in ankylosing spondylitis: A comparison of percutaneous and open techniques

**DOI:** 10.1186/s13018-022-03378-w

**Published:** 2022-11-24

**Authors:** JingYao Ye, Ping Jiang, HuaPeng Guan, ChuanFu Wei, Sen Li, MengLong Jia, NianHu Li

**Affiliations:** 1 Department of Orthopaedics, Shandong University of Traditional Chinese Medicine, Jinan, China; 2 Department of Orthopaedics, Affilited Hospital of Shandong University of Traditional Chinese Medicine, Jinan, China; 3 Department of Orthopaedics, Shanghai University of Traditional Chinese Medicine, Shanghai, China; 4 Department of Orthopaedics, Department of Rheumatology, Shanghai Guanghua Hospital of Integrated Traditional Chinese and Western Medicine, Shanghai University of Traditional Chinese Medicine, Shanghai, China; 5 Department of Orthopaedics, Weifang Hospital of Traditional Chinese Medicine, Weifang, China

**Keywords:** Ankylosing spondylitis, Thoracolumbar fracture, Percutaneous internal fixation, Posterior pedicle screw–rod system, 3D printing, Intraoperative neurophysiological monitoring

## Abstract

**Background context:**

Posterior percutaneous long-segment internal fixation and open fixation with long-segment screws have been used to treat thoracolumbar fractures in ankylosing spondylitis patients.

**Purpose:**

To observe the clinical effect of posterior percutaneous long-segment internal fixation in 26 ankylosing spondylitis (AS) patients with thoracolumbar fractures.

**Study design:**

Retrospective cohort study.

**Patient sample:**

Forty-seven AS patients who were diagnosed with thoracolumbar fractures and treated from December 2014 to December 2018.

**Outcome measures:**

Visual analog scale score, Cobb angle, American Spinal Injury Association Grade, SF-Qualiveen score, pedicle screw misplacement rate, operative duration, blood loss, complications, bed rest duration and modified MacNab score.

**Methods:**

All patients were divided into the percutaneous group (PG) and the open group. Twenty-six patients were treated with percutaneous long-segment internal fixation, and the remaining 21 underwent open fixation with long-segment screws. The minimum follow-up period was 12 months.

**Results:**

The operations were successful in both groups. A patient in the PG showed class C wound healing, while the others showed class A healing, and some patients experienced perioperative complications. All patients were followed up for 12–48 months (mean, 33.81 months), and all patients showed clinical osseous fracture healing. Significant differences were found in operative duration, intraoperative blood loss and postoperative bed rest duration between the two groups (*P* < 0.05). No significant difference was found in improvement of the visual analog scale score, Cobb angle of spinal kyphosis or neurological function after the operation (*P* > 0.05).

**Conclusions:**

As a minimally invasive procedure, posterior percutaneous long-segment internal fixation requires less time, results in less blood loss and causes less trauma. This procedure can also improve patients’ pain, neurological function and kyphotic deformity and achieve effects similar to those of traditional methods. With this curative clinical effect, this procedure can be used as an ideal surgical treatment for thoracolumbar fractures in AS patients, especially for elderly patients with underlying diseases and high surgical risk.

## Introduction

Ankylosing spondylitis (AS) is a disease that mainly involves the axial joints and bilateral sacroiliac joints and causes characteristic inflammatory back pain, which easily leads to structural and functional disorders and decreases quality of life [1]. Approximately 25% of AS patients with a disease duration of 10 years suffer from osteoporosis, and vertebral fractures occur in approximately 10% of these patients [2]. Related studies have shown that the probability of fractures in AS patients is much higher than that in healthy people, especially thoracolumbar fractures (most of which are stress fractures) [3]. AS patients often develop ossification of soft tissue lesions which causes thoracolumbar protrusion deformities, osteoporosis, spinal stiffness, “bamboo spine,” and increased bone fragility, such that even minor external force can cause three-column spinal fractures, which are associated with not only significant instability but also spinal cord nerve injury, and serious potential consequences [4–6]. TLFAS can be treated surgically and nonsurgically. Nonsurgical treatment is safer than surgical correction (due to its high postoperative morbidity and mortality rates) [7]. However, nonsurgical treatment cannot achieve absolute immobilization and often causes fracture nonunion and secondary nerve injury, while long-term bed rest easily leads to complications such as bedsores, pneumonia and venous thrombosis [8]. Therefore, with the advent of modern spinal internal fixation, TLFAS is treated with surgery. To the best of our knowledge, at present, there is a lack of research that compares the merits of percutaneous and open surgical strategies through long-term follow-up. In this study, we analyzed the long follow-up results of patients with posterior percutaneous long-segment internal fixation compared with those who underwent open fixation with long-segment screws for TLFAS, observed the clinical efficacy and provided a reference for the selection of clinical treatment methods in the future.

## Materials and methods

### Study design

This was a retrospective cohort study from a single institution. According to the study design and national and institutional guidelines, ethics committee approval was not needed. At the time of hospitalization, all patients provided written informed consent for surgery and data management for scientific purposes. This study complies with the World Medical Association Declaration of Helsinki.

### Diagnostic criteria

AS was diagnosed when any of the radiological criteria or any of the clinical criteria were present [9].

### Inclusion and exclusion criteria

#### Eligibility criteria

1. AS according to the 1984 mNY;
2. disease duration of at least 5 years;
3. computed tomography (CT) or magnetic resonance imaging (MRI)-confirmed thoracolumbar fracture; and
4. treatment with posterior long-segment fixation after informed consent was obtained from patients and their families.

#### Exclusion criteria

1. another severe concomitant disease and an inability to undergo surgery;
2. multiple concomitant fractures;
3. another concomitant spinal disease (e.g., spinal tuberculosis, pyogenic myelitis or spinal tumor);
4. an allergic constitution or a psychological disorder;
5. conservative treatment or external fixation therapy; and
6. follow-up period of less than 12 months.

### Treatment protocol

*Surgical selection criteria* Recently, with the popularity of percutaneous internal fixation [10], the surgical group, after mastering the percutaneous technology, chose percutaneous internal fixation for patients who do not need decompression or who need decompression but cannot tolerate open surgery through preoperative evaluation.

The pedicle screw–rod system used in the treatment was provided by the same company. Patients in both groups were treated with antibiotics 30 min before the operation to prevent infection. All patients were placed under general anesthesia in the prone position. In the process of body positioning, attention should be given to reducing or preventing fracture opening. All surgical treatments were performed by the same surgical team.

### Percutaneous long-segment internal fixation

C-arm fluoroscopy was performed to identify the affected vertebral segment and the projection bias of the two vertebral pedicles on the body surface. On both sides of the spine, 1.5 cm away from the midline, the needle puncture sites were marked, and conventional disinfection and draping with sterile towels were performed. At the puncture sites, a needle of an appropriate size was advanced into the anterior 1/3, and then, the position of the needle was evaluated. Retractors were used to expand the openings for the implantation of appropriately sized pedicle screws. Then, satisfactory positioning of the screws was verified, which is especially important in patients with preoperative nerve or spinal cord injury, and decompression of the affected segment was performed. On the superior side of the vertebral body, two titanium rods were connected to the pedicle screws. After confirmation of appropriate positioning, the incision was irrigated, hemostasis was achieved, and the incisions were sutured.

### Open fixation with long-segment screws

The surgical procedure is similar to the previous surgical method. Then, a midline incision centered on the affected vertebral body was made and was approximately 25–30 cm in length. For those with neural (or spinal cord) compression, appropriate decompression was needed [11].

### Postoperative management

Patients in both groups were given routine postoperative care and treatment. Patients were also encouraged to perform straight-leg exercises and dorsiflexion and plantar flexion of the ankle. Postoperatively, patients in both groups used a thoracolumbar brace for 3 months.

### Outcomes and measurements

The clinical outcomes were evaluated by data collected using questionnaires. Pre- and intraoperative information was extracted from patients’ medical files, including trauma types and mechanisms (low- or high-energy trauma). Screw misplacement was independently evaluated by two professionals who were not involved in the study on intraoperative and postoperative CT scans and classified according to the grading system of Gertzbein and Robbins [12]. The operative duration, intraoperative blood loss, postoperative bed rest duration and hospitalization duration were recorded, and the rate of pedicle screw misplacement, as determined by intraoperative C-arm fluoroscopy, i.e., the screw was not inserted through the pedicle, was calculated. The following clinical variables were recorded at hospital admission (baseline) and at follow-up for assessment of the overall treatment outcome: the VAS score, ASIA grade (A–E) and modified MacNab score. All patients were assessed by telephone or outpatient follow-up at 6 and 12 months. The fracture healing line, incidence of spinal cord injury and Cobb angle of kyphosis were evaluated by CT and MRI.

### Statistical analysis

Data were statistically analyzed using SPSS (version 26.0, IBM, USA). Categorical variables are presented as counts with percentages, and continuous variables with a normal or nonnormal distribution are presented as the mean ± standard deviation or median (range), respectively. The chi-square (*χ*^2^) test, the *t* test or nonparametric tests were used to assess differences among approaches, as appropriate. Unless stated otherwise, a two-tailed *P* value of less than 0.05 was considered statistically significant.

## Results

### General information

A total of 47 patients were enrolled and divided into the percutaneous group (PG) and the open group (OG). The most frequent mechanism of injury was a ground level fall in 12 patients (46.15%) in the PG group and 11 patients (52.38%) in the OG group. Nine patients (34.62%) in the PG group and 2 patients (9.53%) in the OG group incurred fractures without any distinct trauma. The other mechanism of injury was car or motorbike accidents: 5 patients (19.23%) in the PG group and 8 patients (38.10%) in the OG group. Fractures classified by the AO classification were B1 in 14 (53.85%), B3 in 7 (26.92%) and C in 5 (19.23%) patients in the PG group; B1 in 10 (47.62%), B3 in 7 (33.33%) and C in 4 (19.05%) patients in OG group. At baseline, there were no significant differences in terms of sex, age, smoking, BMI, medical diseases, history of AS, VAS score, Cobb angle, ASIA grade or SF-Qualiveen score (*P* > 0.05) (Table 1).

**Table 1:** Case information for the two groups

	PG (26)	OG (21)	*P* value
*Sex*			0.505
M	21	15	
F	5	6	
Age	29–85 (58.12 ± 15.72)	35–86 (55.43 ± 14.83)	0.553
Smoking	10	13	0.110
BMI	20.96–29.98 (24.58 ± 2.33)	19.37–28.98 (24.57 ± 2.33)	0.983
Presence of medical diseases	15	10	0.491
History of AS	7–50 (21.85 ± 11.00)	6–51 (25.95 ± 10.78)	0.174
VAS score	7.35 ± 1.02	7.38 ± 1.18	0.908
Cobb angle	26.25 ± 12.64	25.77 ± 12.35	0.896
SF-Qualiveen score	5.81 ± 2.14	5.90 ± 2.05	0.875
*ASIA grade*			0.818
B	4	3	
C	3	4	
D	6	4	
E	13	10	
*Traumatic mechanism*			
Ground level fall	12 (46.15%)	11 (52.38%)	
Without any distinct trauma	9 (34.62%)	2 (9.53%)	
Car or motorbike accidents	5 (19.23%)	8 (38.10%)	
AO fracture classification B1	14 (53.85%)	10 (47.62%)	
B2	7 (26.92%)	7 (33.33%)	
C	5 (19.23%)	4 (19.05%)	

### Perioperative complications

The operations were successful in both groups. One patient achieved class C wound healing in the PG, while the other patients achieved grade A. There were no cases of surgical positioning error, pedicle screw positioning error or iatrogenic nerve injury (misplacement rate = 0). However, some patients experienced perioperative complications, and there were 6 perioperative complications in the OG, including 2 cases of DVT of the lower extremities, 2 cases of postoperative anemia, 1 case of postoperative bronchitis and 1 case of intraoperative fracture displacement. In the PG, there were only 2 perioperative complications, including 1 case of high intraoperative blood loss and 1 case of peri-implant infection.

### Clinical outcomes

**Fig. 1:**
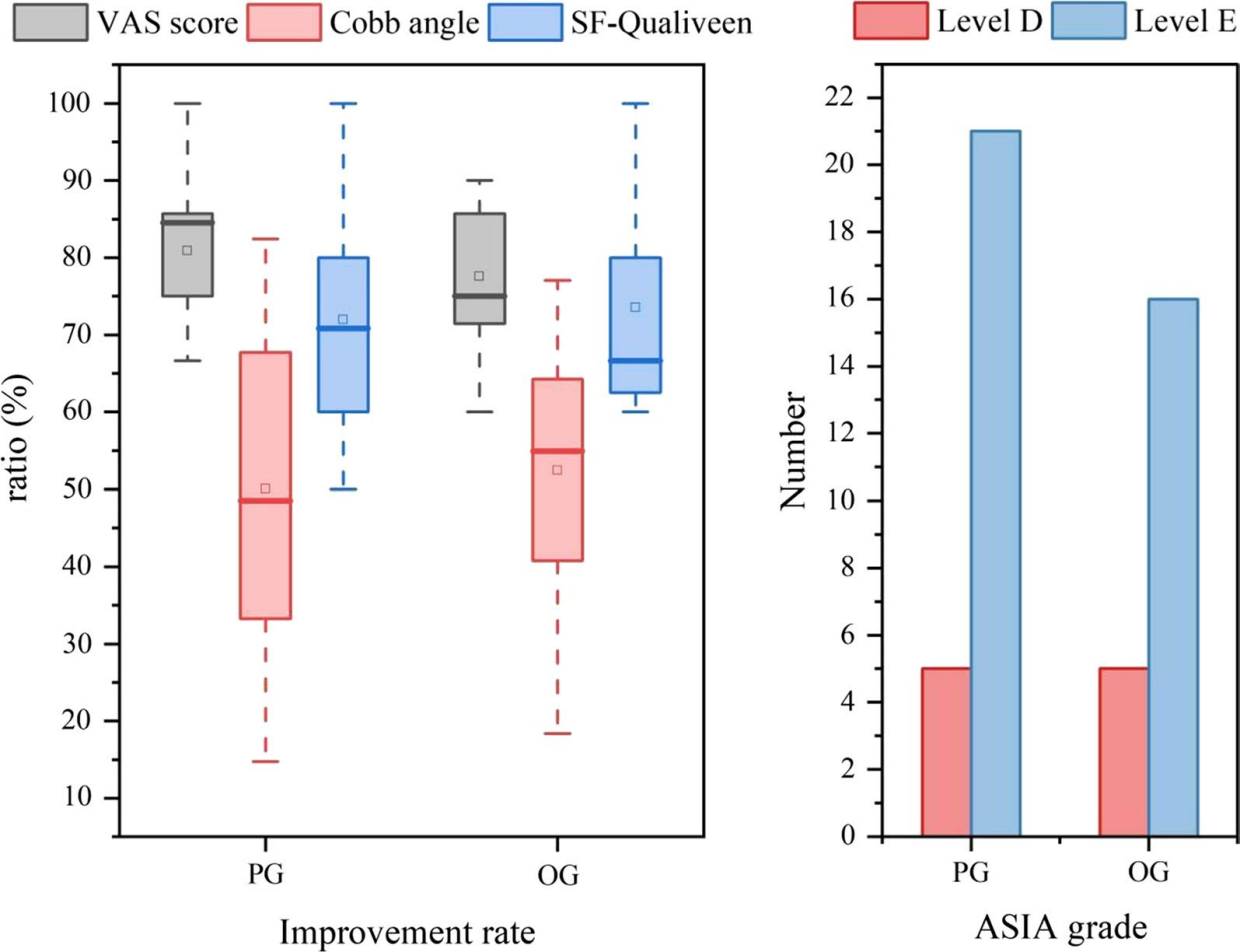
Comparison of postoperative recovery outcomes

**Fig. 2:**
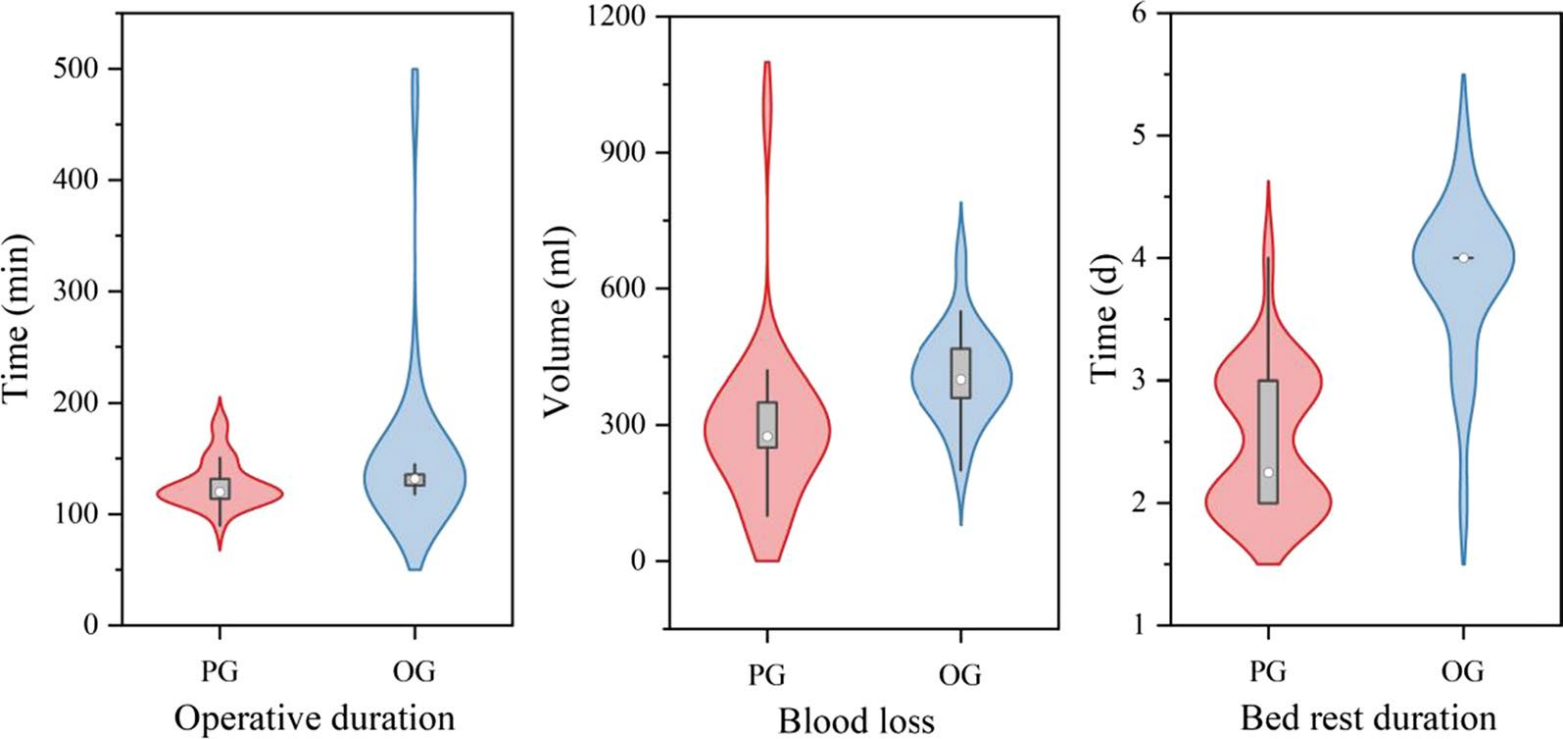
Comparison of perioperative data

Surgery was successfully performed in all patients in both groups. Patients were followed up for 12–48 months, with a mean follow-up of 33.81 months, and all patients showed clinical bony healing of the fractures. The VAS score and Cobb angle showed significant improvement postoperatively compared with preoperatively, and there was no significant difference in the scores, improvement rates or incidence of perioperative complications between the two groups (*P* > 0.05). The SF-Qualiveen scale and the ASIA score were not significantly different between the two groups (*P* > 0.05). The perioperative parameters in the PG were as follows: operative duration, 90–180 (127.27 ± 21.17) min; intraoperative blood loss, 50–1000 (292.15 ± 177.66) ml; bed rest duration, 2–4 (2.52 ± 0.57) days; and perioperative complications, 2 cases. The perioperative parameters in the OG were as follows: operative duration, 118–480 (153.29 ± 78.10) min; intraoperative blood loss, 200–670 (409.81 ± 101.47) ml; bed rest duration, 2–5 (3.83 ± 0.64) days; and perioperative complications, 6 cases. Comparing the data between the two groups, the patients in the PG group had significant advantages (*P* < 0.05) over the patients in the OG group in terms of the operative duration, blood loss and bed rest duration. Using the modified MacNab score to assess the postoperative results, patients in both groups were satisfied with the surgical efficacy, and the rate of an excellent outcome was ≥ 90%, with no statistically significant difference between the groups (*P* > 0.05) (Table 2, Figs. 1 and 2).

**Table 2:** Comparison of various parameters between the PG group and the OG group

	PG (26)	OG (21)	*P* value
VAS score (6 months)	2.46 ± 0.91	2.62 ± 0.87	0.475
VAS score (1 year)	1.42 ± 0.58	1.62 ± 0.50	0.249
Improvement rate (%)	80.90 ± 7.51	77.56 ± 7.94	0.204
Cobb angle (6 months)	13.68 ± 10.04	11.75 ± 6.43	0.700
Cobb angle (1 year)	13.45 ± 9.99	11.53 ± 6.38	0.716
Improvement rate	50.05 ± 19.89	52.42 ± 16.34	0.663
SF-Qualiveen score	1.81 ± 1.20	1.67 ± 1.07	0.782
Improvement rate (%)	71.99 ± 14.66	73.55 ± 13.35	0.796
*ASIA grade*			0.566
D	4	5	
E	8	6	
No change	14	10	
Improvement rate (%)	33.33	66.67	0.486
Recovery rate (%)	45.45	54.55	0.870
Perioperative complications	2	6	0.115
Operative duration (min)	127.27 ± 21.17	153.29 ± 78.10	0.007
Blood loss (ml)	292.15 ± 177.66	409.81 ± 101.47	< 0.001
Bed rest duration (d)	2.52 ± 0.57	3.83 ± 0.64	< 0.001
*Modified MacNab score*			0.969
Excellent	17	14	
Good	7	5	
Average	2	2	
Poor	0	0	
Improvement rate (%)	92.31	90.48	0.610

## Discussion

Ankylosing spondylitis of the spine causes the biomechanical structure of the spine to change such that the whole spine gradually fuses and becomes more similar to a long bone [13]. High frequency and occult fracture, vertebral osteoporosis, stiffness and kyphosis are common in this group [8, 14]. Fractures are usually associated with low-energy trauma [15]. Heterotopic bone fracture may be due to hematoma or oppressive nerve block in different degrees of neurological symptoms [16–18]. For patients with unstable spine fracture and spinal cord injury or hematoma AS patients, surgery is often recommended. In this study, according to the clinical manifestations and imaging examination findings, the surgeon performed local decompression on patients with bone block invasion of the spinal canal of more than 4 mm or patients with a preoperative ASIA score indicating the need for local decompression at level 2 or 3, and the neurological symptoms of these patients were improved to varying degrees after surgery.

At present, it is believed that spinal fractures in patients with AS should be treated according to the principles of treating long bone fractures, and there are three main surgical approaches for treating TLFAS: the anterior approach, posterior approach and combined anterior and posterior approach [19]. We prefer the posterior approach. With minimally invasive technology gradually being applied in surgical operations, in elderly patients who are at high risk in routine operations, doctors may apply minimally invasive methods to reduce the risks associated with the operation and its related complications; such methods include minimally invasive percutaneous pedicle screw fixation (MIPPSF) technology, which has gradually emerged in the field of spinal fracture treatment in recent years. Through a retrospective study of the clinical data of 47 patients with TLFAS treated by percutaneous and conventional posterior internal fixation, it was found that there was no significant difference in clinical efficacy between the two surgical methods (Table 2, Fig. 1). However, the patients in the PG group showed significant advantages over the patients in the OG group in terms of the operative duration, blood loss and bed rest duration (Table 2, Fig. 2). In this study, there was insufficient evidence to demonstrate a difference in perioperative complications between the two surgical methods, possibly due to the small sample size; however, the result (*P* = 0.115) indicated a trend of a difference.

**Fig. 3:**
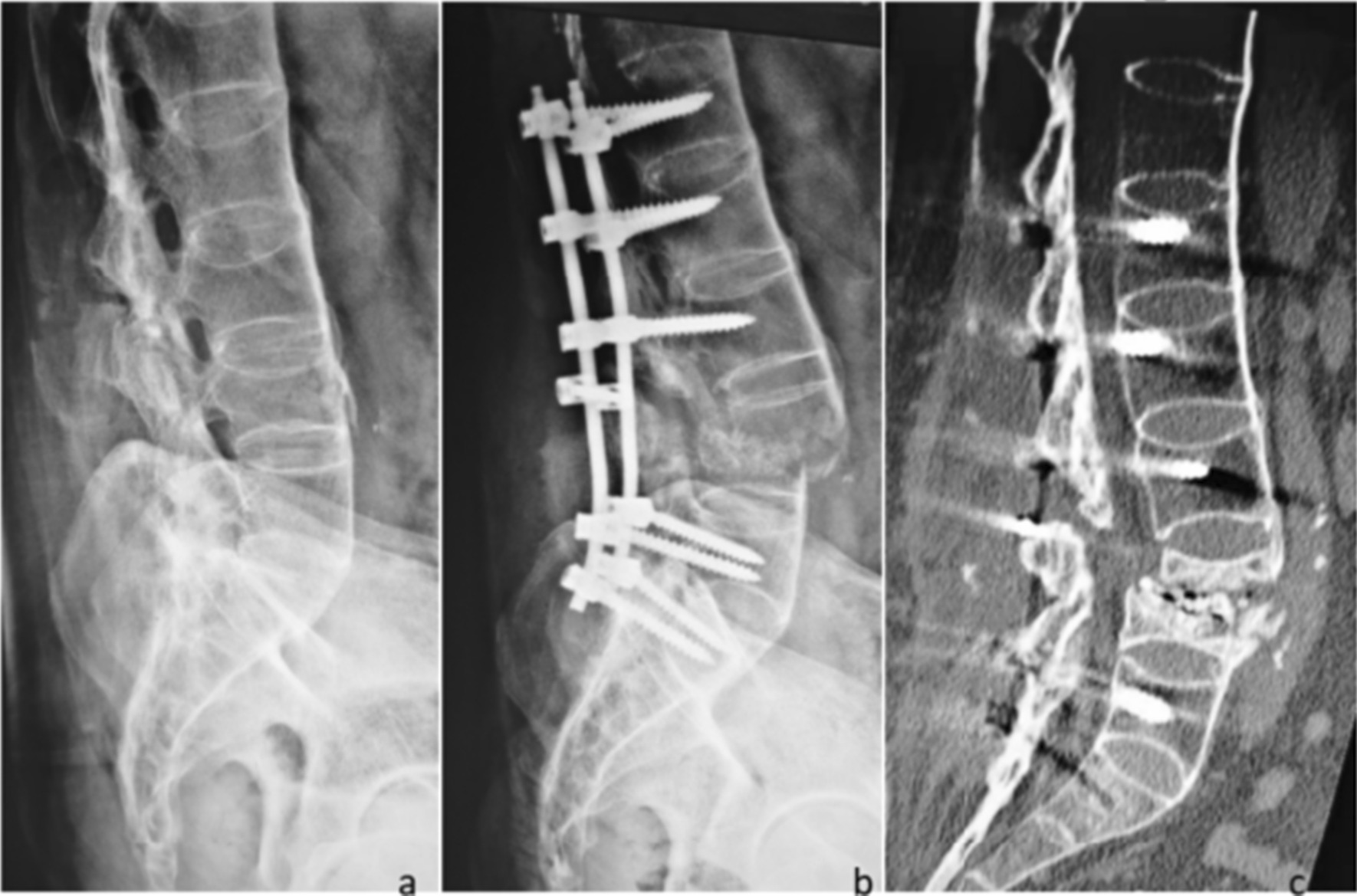
Complication 1, Fracture displacement in a 42-year-old male in the OG group. Fracture displacement occurred during preoperative reduction. The patient was closely observed during and after surgery and showed no neurological impairment, and smooth bone healing was observed. **a**: Preoperative image. **b**: Postoperative image. **c**: Image obtained at 3 months postoperatively

**Fig. 4:**
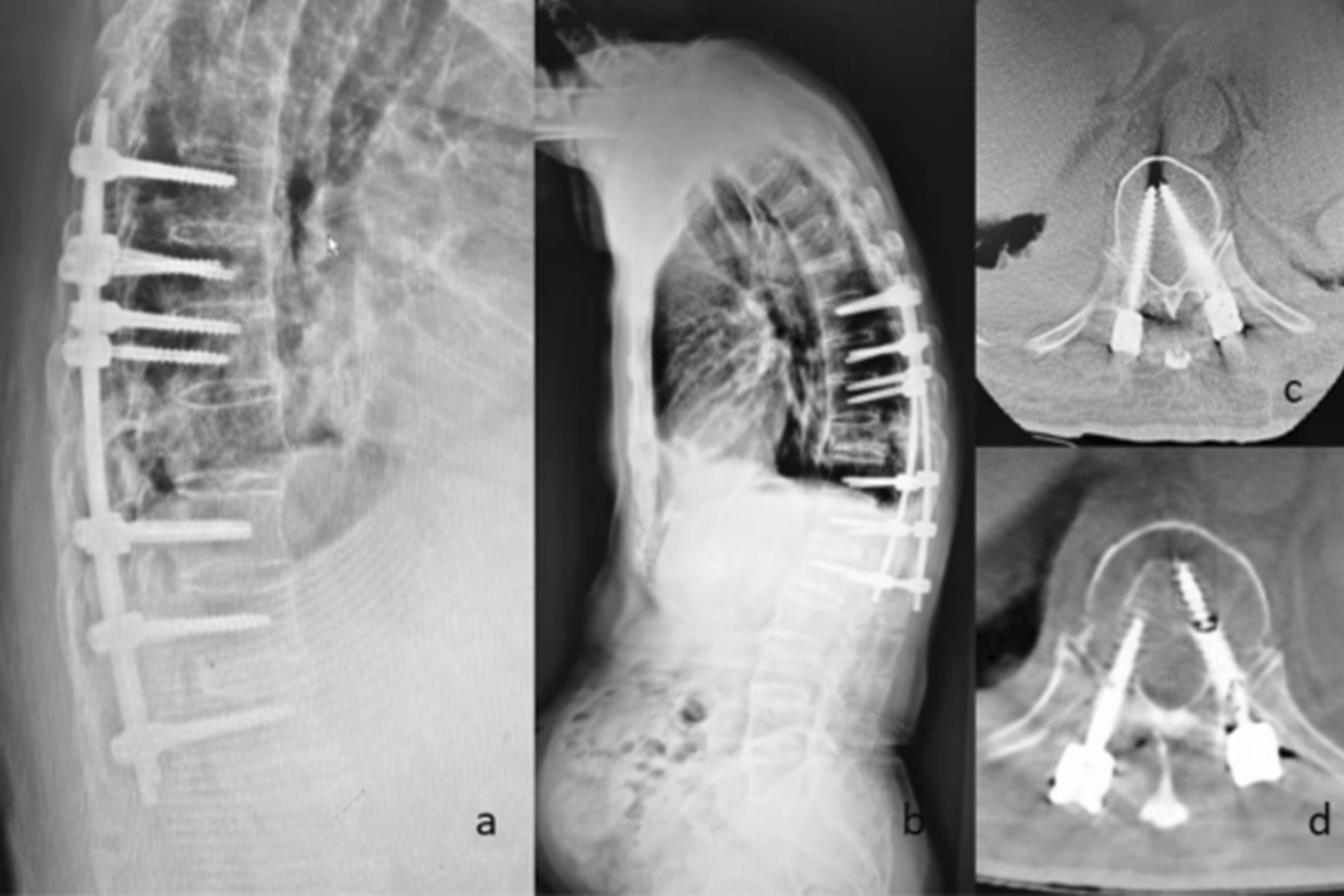
Complication 2, Screw loosening in an 85-year-old male in the PG group. The patient engaged in intense exercise after surgery, resulting in screw loosening, without pain or neurological impairment. **a**, **c**: Images obtained on the fourth day after surgery. **b**, **d**: Images obtained at 7 months after surgery

In this study, 6 patients in the OG group had perioperative complications, and these results are consistent with those of previous studies showing that greater surgical trauma increases the risk of complications [20]. The other patient with a perioperative complication (complication 1, Fig. 3) had fracture displacement, which occurred during preoperative reduction and was unrelated to the surgical method. Percutaneous internal fixation was replaced with open surgery to avoid further nerve damage during the operation. Osteoporosis needs to be considered in patients with AS, and the fracture displacement in this case was likely due to aggressive manipulation, which suggested that surgeons should avoid aggressive manipulation in the pursuit of anatomical reduction and exert caution [21], observe the fracture under C-arm fluoroscopy and perform bioelectric detection in real time during reduction. Meanwhile, in the PG group, pedicle screws can easily damage the blood vessels of AS patients with osteoporosis, and it can be difficult to stop subsequent bleeding due to the small amount of exposure. In this case, bone wax was used to stop the bleeding, and the hollow channels inside the screws were filled with medical bone wax for complete hemostasis. The clinical effect was good in most cases, but the effect was poor in this patient. Meanwhile, the postoperative infection that occurred in the PG group was also unrelated to the surgical method and may have been caused by improper intraoperative techniques or postoperative nursing care, which suggested the need for standardized operating procedures and improved education regarding postoperative care [22]. In addition to the above perioperative complications, one patient in the PG group was found to have screw loosening at the 7-month follow-up (complication 2, Fig. 4), and the patient was found to have participated in strenuous postoperative activity. This case suggests that in AS patients with osteoporosis, strenuous activity should be avoided after surgery. In addition, if the patient is found to have very severe osteoporosis, i.e., a bone mineral density (BMD) less than 3.5, we will select a cannulated screw system and inject an appropriate amount of bone cement to form a bone cement-vertebral pedicle screw-strengthening system, which will help increase the grip strength of the implant and prevent postoperative screw loosening. Although some studies have proven that exercise or global postural reeducation (GPR) is beneficial in improving the pain and physical function of AS patients [23, 24], for patients with TLFAS, especially elderly patients, avoiding strenuous activity is still recommended to prevent screw loosening or even refracture.

**Fig. 5:**
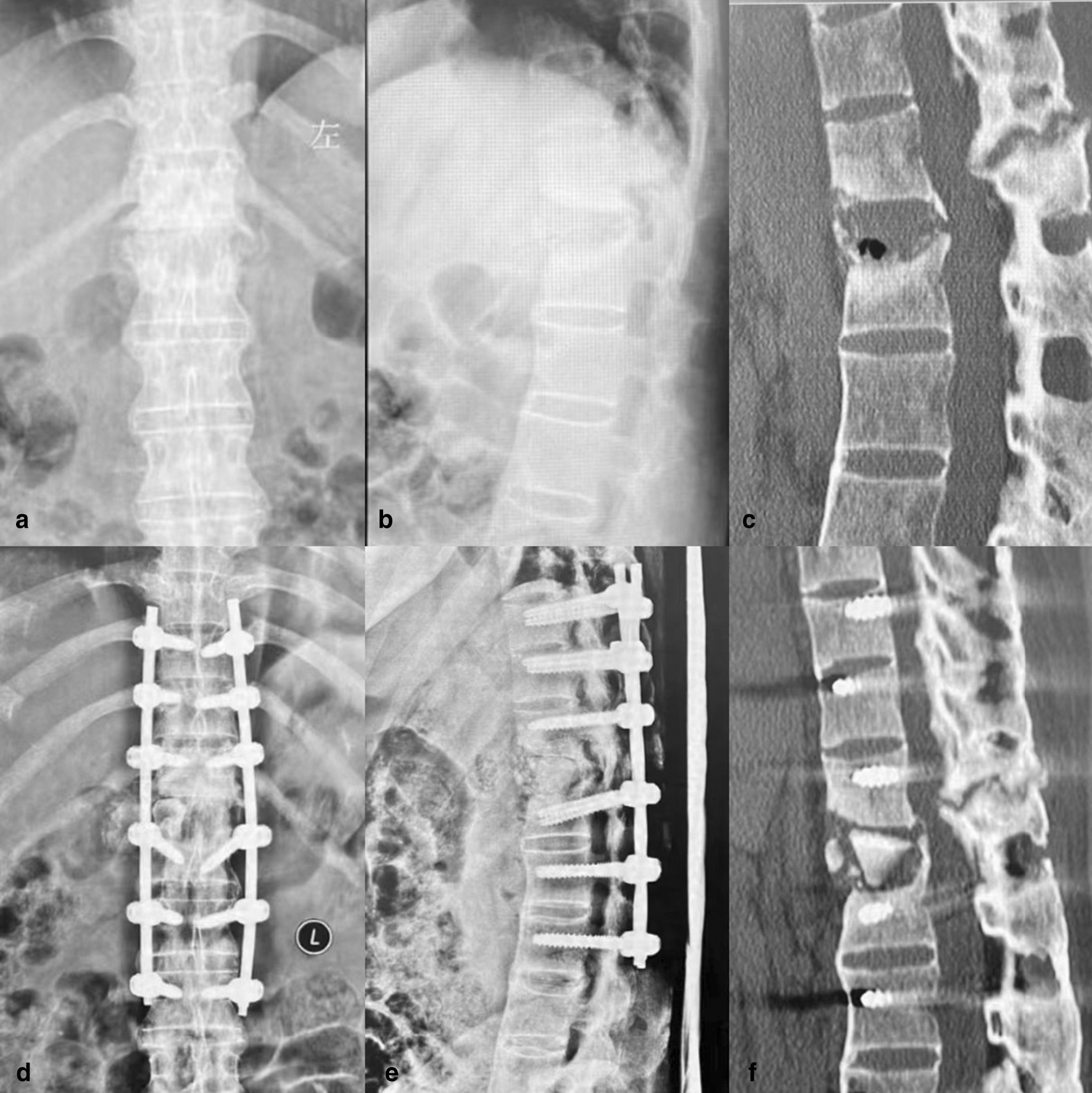
Typical case 1: Improved percutaneous screw placement in a 44-year-old female in the PG group, with a history of AS for 10 years and thoracolumbar fractures (T12, L1). **a**–**c**: Preoperative images showing fractures of T12 and L1. **d**–**f**: Images on the second day after the operation showing good positioning of the instrumentation

By retrospectively studying the complications of these two surgical methods, it can be seen that percutaneous internal fixation through one point for each screw, corresponding to a small skin incision less than 1 cm, does not require a large degree of exposure and reduces the damage to the deep layers of muscle and fascia, thereby reducing the intraoperative trauma, postoperative pain, recovery time, bed rest duration and incidence of perioperative complications [25]. Patients who have preoperative spinal cord damage or nerve damage are also suitable for percutaneous internal fixation. For these patients, we can first perform percutaneous long-segment fixation and then facilitate decompression of the spinal cord or autologous bone block implantation in the cavity through another small incision of approximately 5 cm (Fig. 5, typical case 1). This modified method not only has the advantages of minimal trauma but also allows decompression and bone block implantation to be achieved. However, according to our experience, percutaneous internal fixation is more suitable for AS patients with no neural spinal cord compression or dislocated fractures. According to personal opinion, achieving strong internal fixation and reducing the trauma and the risk of surgery should be prioritized over achieving neural spinal cord compression. However, we have limited experience in the use of percutaneous internal fixation for the treatment of TLFAS, and these opinions may change with the accumulation of experience.

**Fig. 6:**
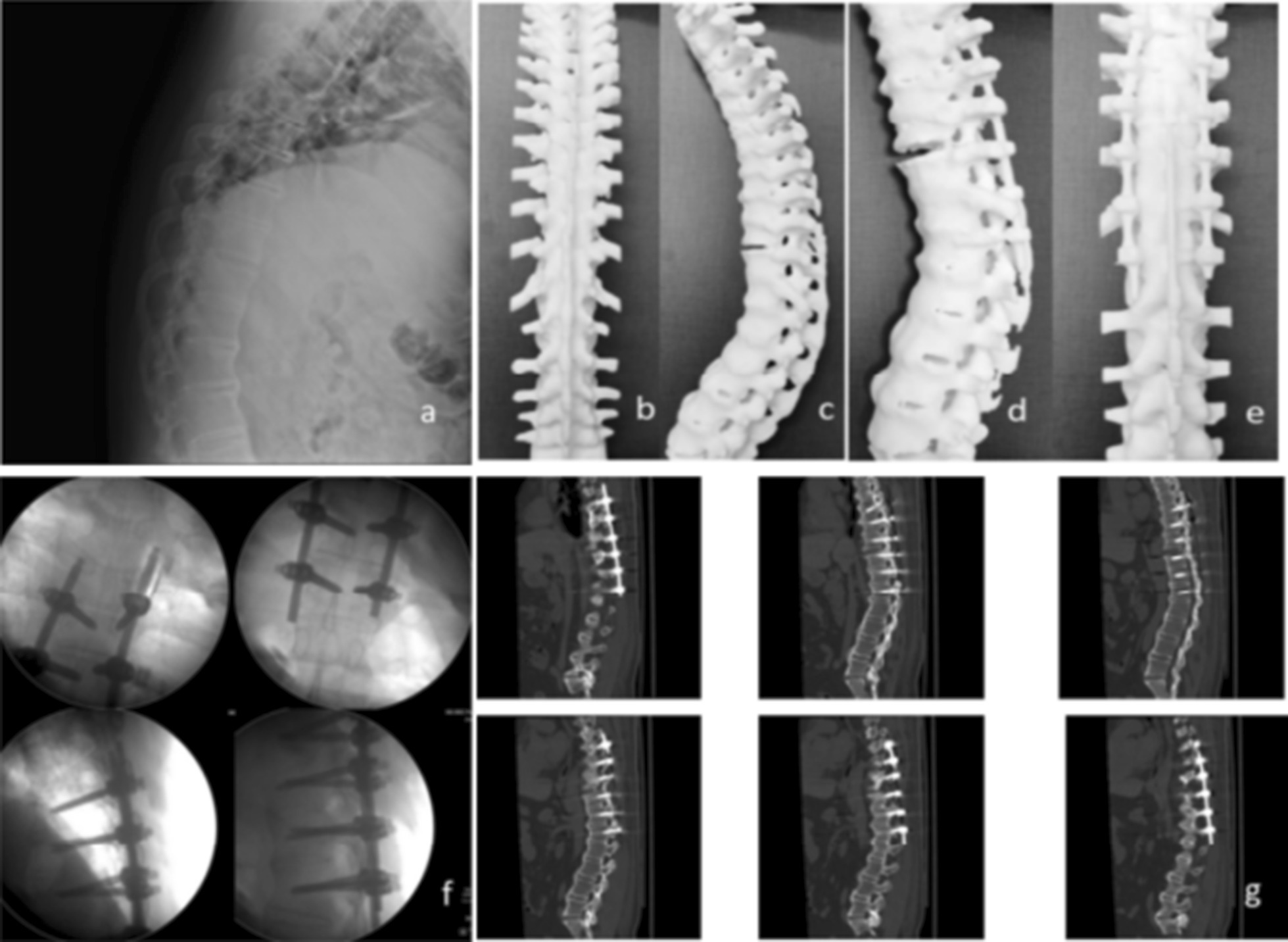
Typical case 2: Case of 3DP-RP applied in a 37-year-old male with AS and a thoracic fracture (T11) in the PG group. **a**: Preoperative sagittal X-ray. **b**, **c**: 3DP-RP models manufactured after injury. **d**, **e**: 3DP-RP models produced after simulating the surgery. **f**: Images obtained intraoperatively showing successful placement of the screws and rods. **g**: Postoperative CT images showing ideal positioning of the screws and rods

In addition, we speculate that other patients with spinal ankylosis, such as those with diffuse idiopathic skeletal hyperostosis (DISH), can also be treated with this method when presenting with thoracolumbar fractures. With the widespread clinical application of percutaneous internal fixation technology, the problems of this technology can be summarized and analyzed. In minimally invasive methods, small incisions provide many benefits but also increase the difficulty of the procedure for surgeons. Particularly in long-segment fixation, the titanium rods are longer and not easy to control during bending, and the field is relatively narrow and further from the spine, which may lead to increased intraoperative blood loss, a prolonged operation, reduced screw accuracy, screw loosening or even screw pullout. Because spinal lesions in patients with AS lead to segmental kyphosis, an abnormal posterior column structure, it is difficult to effectively identify abnormal anatomical signs during pedicle screw implantation, such as facet joint abnormalities and the “human crest” sign [26], especially in some obese, bulky patients [27]. This increases the difficulty of quickly identifying the point and direction of insertion, so the surgeon can only rely on C-arm fluoroscopy and clinical experience. The operation is difficult, the learning curve is long, and the technical requirements of the operator are high. To avoid the above problems, we used 3D printing and rapid prototyping (3DP-RP) technology (Fig. 6, typical case 2). Using 3DP-RP for preoperative surgical planning and placing pedicle screws according to the predetermined entry point and direction during the operation not only facilitated rod placement but also improved the accuracy of pedicle screw implantation [28] and reduced blood loss, nerve injury and the duration of the operation. At the same time, this technique reduced the number of intraoperative fluoroscopies and the radiation exposure of surgeons and patients. Additionally, 3DP-RP facilitates preoperative patient education and communication [29] and can significantly relieve patients’ anxiety-related pain [30, 31]. Moreover, spinal restoration is very important, and good restoration is conducive to titanium rod placement and Cobb angle correction. Good restoration can be achieved by postural reduction before the operation [15]. The quality of restoration will directly affect the efficiency and effect of rod placement. Intraoperative neurophysiological monitoring (IONM) can be used to detect nerve damage by monitoring neural pathways during the operation. Intraoperative monitoring for stress, injury or neuropathy allows the extent of the lesion to be determined and real-time operations to be performed by auxiliary surgeons to prevent identification of the affected nerves and prevent further damage, mitigating the risk of nerve injury [32]. Overall, this approach improves the long-term prognosis of motor function and reduces the incidence of both spinal cord nerve injury and postoperative complications [33]. After achieving satisfactory spinal positioning, the nut of the middle screw should be tightened first, proceeding with tightening toward the two ends; this approach prevents the middle titanium rod from warping when the nuts at the two ends are tightened first. The results of this study may be affected by the causes of injury in the two groups, and the different degrees of injury may affect the prognosis of patients, which would be difficult to solve.

In conclusion, percutaneous internal fixation has the advantages of minimal trauma and quick recovery and is worthy of clinical application. In addition, personalized treatment for each patient will be the direction of spinal surgeons’ future efforts to improve quality of life and reduce costs.

## Data Availability

The datasets used or analyzed during the current study are available from the corresponding author on reasonable request.
